# Possible Human-to-Dog Transmission of SARS-CoV-2, Italy, 2020

**DOI:** 10.3201/eid2707.204959

**Published:** 2021-07

**Authors:** Nicola Decaro, Gabriele Vaccari, Alessio Lorusso, Eleonora Lorusso, Luca De Sabato, Edward I. Patterson, Ilaria Di Bartolo, Grant L. Hughes, Liana Teodori, Costantina Desario, Barbara Colitti, Dominga Ricci, Domenico Buonavoglia, Sergio Rosati, Vito Martella, Cesare Cammà, Umberto Agrimi, Gabriella Elia

**Affiliations:** University of Bari, Valenzano, Italy (N. Decaro, E. Lorusso, C. Desario, D. Buonavoglia, V. Martella, G. Elia);; Istituto Superiore di Sanità, Rome, Italy (G. Vaccari, L. De Sabato, I. Di Bartolo, U. Agrimi);; Istituto Zooprofilattico Sperimentale dell’Abruzzo e del Molise “G. Caporale,” Teramo, Italy (A. Lorusso, L. Teodori, C. Cammà);; Liverpool School of Tropical Medicine, Liverpool, UK (E.I. Patterson, G.L. Hughes);; University of Turin, Turin, Italy (B. Colitti, S. Rosati);; Ambulatorio Veterinario Dott.ssa Ricci Dominga, Andria, Italy (D. Ricci)

**Keywords:** dogs, humans, SARS-CoV-2 transmission, next-generation sequencing, phylogeny, SARS-CoV-2, COVID-19, respiratory infections, severe acute respiratory syndrome coronavirus 2, 2019 novel coronavirus disease, coronavirus disease, zoonoses, viruses, coronavirus, Italy

## Abstract

We detected severe acute respiratory syndrome coronavirus 2 in an otherwise healthy poodle living with 4 family members who had coronavirus disease. We observed antibodies in serum samples taken from the dog, indicating seroconversion. Full-length genome sequencing showed that the canine and human viruses were identical, suggesting human-to-animal transmission.

Coronavirus disease (COVID-19), caused by infection with severe acute respiratory syndrome coronavirus 2 (SARS-CoV-2), emerged in humans in Wuhan, China, in late December 2019, probably because of spillover from an unidentified animal host (*1*). Dogs and cats, to which some coronaviruses are endemic (*2*), are also susceptible to SARS-CoV-2 infection (*3*,*4*). Although the spread of SARS-CoV-2 is maintained mainly by human-to-human transmission, the epidemiologic implications of animal susceptibility remain uncertain (*4*). We characterized the full genome of a SARS-CoV-2 isolate detected in a dog. 

A female poodle, who was 1.5 years of age, lived with 4 family members in Bitonto, Italy. All family members had signs and symptoms of COVID-19, the illness caused by SARS-CoV-2 infection. High temperature (37.5°C–38.5°C), coughing, anosmia, and ageusia developed in the mother, who was 54 years of age, on October 31, 2020. The woman tested positive for SARS-CoV-2 by a rapid antigen test conducted on November 3, 2020. The local health authority collected nasopharyngeal swab samples and used molecular testing to confirm SARS-CoV-2 infection in the woman’s husband and 2 daughters. Clinical signs in the other family members ranged from mild fatigue and high temperatures (37.5°C–37.8°C) in the daughters to moderate respiratory signs and persistent high temperature (37.8°C–38.6°C) in the husband. This study was approved by the Ethics Committee of the Department of Veterinary Medicine at the University of Bari (approval no. 15/2020). 

On November 4, 2020, the owners collected oral and nasal swab samples from the family’s poodle according to our instructions. The pooled samples tested positive for SARS-CoV-2 by real-time reverse transcription PCR selective for the N gene (*5*). During the next 11 days, the owners collected nasal, oral, and rectal swab samples from the dog. Of 20 samples collected during November 6–15, a total of 4 samples (all of which were collected during November 6–9) tested positive for SARS-CoV-2 (Table). Viral shedding occurred at low titers. We did not isolate the virus. The dog did not show any clinical signs, and no other pets lived in the household.

**Table:** Molecular and serologic testing of dog with severe acute respiratory syndrome coronavirus 2 infection, Italy, 2020*

Date of sample collection	Real-time reverse transcription PCR C_t_ values				Serologic assay results			
	Oral	Nasal	Rectal		ELISA ID.vet†	ELISA In3Diagnostic‡	PRNT_80_§	VNT¶
2020 Nov 4	35.7**	35.7**	ND		ND	ND	ND	ND
2020 Nov 6	ND	37.64	ND		ND	ND	ND	ND
2020 Nov 7	35.61	–	ND		ND	ND	ND	ND
2020 Nov 8	ND	–	40.71		ND	ND	ND	ND
2020 Nov 9	ND	–	36.04		ND	ND	ND	ND
2020 Nov 10	–	–	–		ND	ND	ND	ND
2020 Nov 11	–	–	ND		ND	ND	ND	ND
2020 Nov 12	–	–	ND		ND	ND	ND	ND
2020 Nov 13	–	–	ND		ND	ND	ND	ND
2020 Nov 14	–	–	ND		ND	ND	ND	ND
2020 Nov 15	–	–	ND		ND	ND	ND	ND
2020 Nov 27	ND	ND	ND		–	+ (23%)††	1:80	1:10
2020 Dec 12	ND	ND	ND		–	–	1:80	1:20

*C_t_, cycle threshold; ND, not done; PRNT_80_, 80% plaque reduction neutralization test; VNT, virus neutralization test; –, negative; +, positive. †ID Screen SARS-CoV-2 Double Antigen Multi-species ELISA (ID.vet, https://www.id-vet.com). ‡Eradikit COVID19-Multispecies (In3Diagnostic, http://www.in3diagnostic.com). §Antibody titer expressed as the highest serum dilution with 80% reduction in plaques in inoculated VERO-E6 cells compared with the control.1:20 was the lowest serum dilution tested. ¶Antibody titer expressed as the highest serum dilution preventing the appearance of cytopathic effect in inoculated VERO-E6 cells. 1:10 was the lowest serum dilution tested. **Pooled oral and nasal swab specimens. ††Ratio between the optical densities of the tested serum and the positive control (cutoff value = 20%).

We tested a serum sample collected by the dog’s veterinarian on November 27 with 2 commercial multispecies ELISA tests: ID Screen SARS-CoV-2 Double Antigen Multi-species ELISA (ID.vet, https://www.id-vet.com) and Eradikit COVID19-Multispecies (In3Diagnostic, http://www.in3diagnostic.com). We also conducted a plaque reduction neutralization test (PRNT) (*4*) and a virus neutralization test (VNT) (*6*). We detected antibodies with the Eradikit (23%), PRNT (1:80), and VNT (1:10). We used serologic assays to confirm the presence of antibodies against SARS-CoV-2 in an additional serum sample collected on December 12, 2020; the antibody titers were 1:80 for PRNT and 1:20 for VNT (Table). 

We submitted the positive pooled oral and nasal swab samples from the dog and the oropharyngeal swab sample from the index patient, all of which were collected on November 4, for next-generation sequencing (*7*). Next-generation sequencing obtained total reads of 929,736 with a mean coverage of 4,300× for the index patient and 969,837 with a mean coverage of 1,800× for the dog. Complete genomes were obtained using the pipeline SARS-CoV-2 RECoVERY in the Galaxy public server ARIES (Istituto Superiore di Sanità, https://w3.iss.it/site/aries). The 2 SARS-CoV-2 genomes shared 100% nucleotide identity. The Pangolin COVID-19 Lineage Assigner (https://pangolin.cog-uk.io) and Nextclade (https://clades.nextstrain.org) assigned the sequences to the lineage B.1.177 (denoted by Nextclade as 20A.EU1) in Europe. Phylogenetic analysis confirmed the clustering of the 2 strains within the GV clade and the B.1.177 lineage already detected in Italy (Figure).

**Figure:**
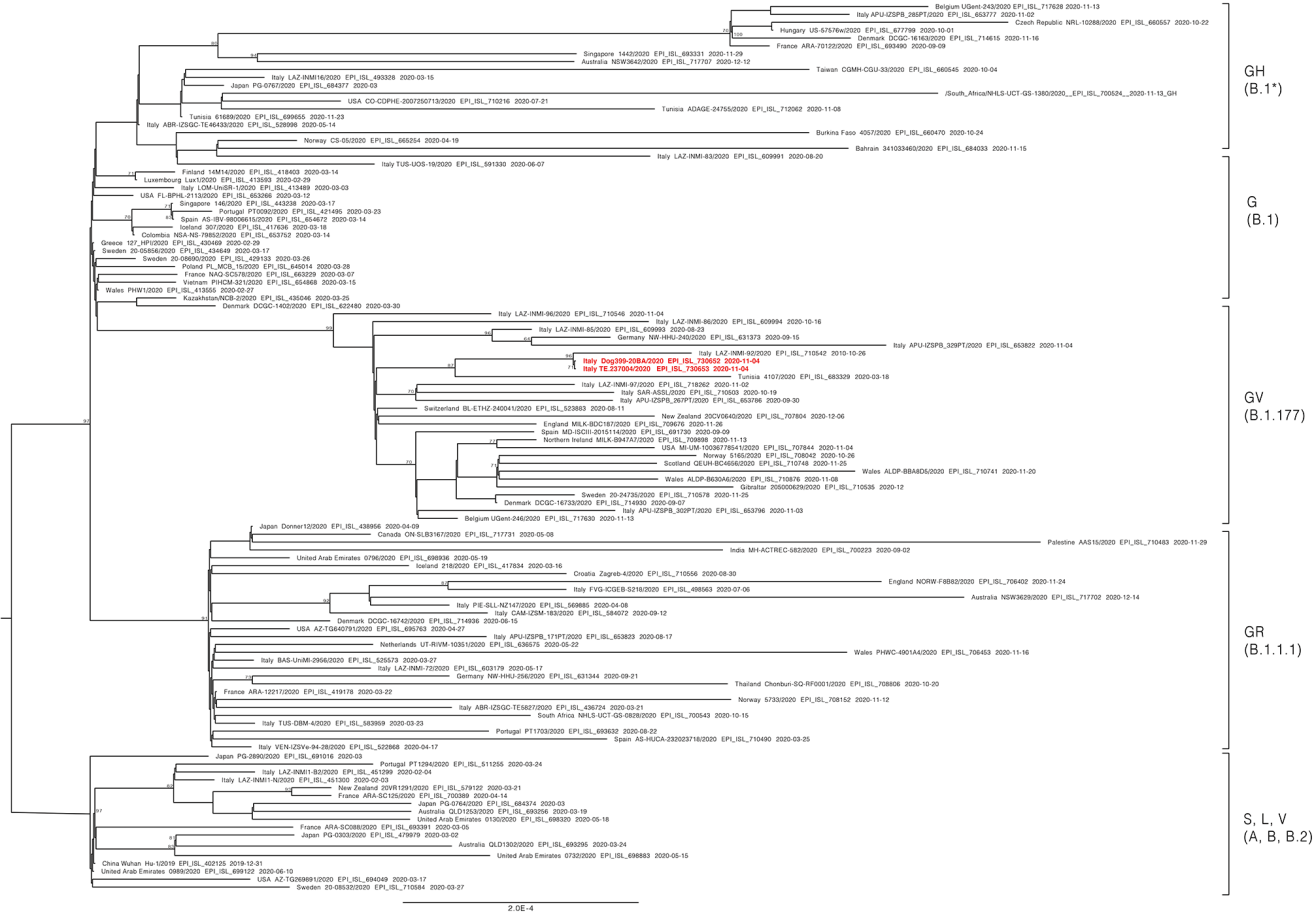
Maximum-likelihood tree comparing 108 strains of severe acute respiratory syndrome coronavirus 2 circulating among humans and canines. Tree shows 107 complete genomes downloaded from the GISAID database (https://www.gisaid.org) and the strains sequenced from an infected dog and family member in Italy (bold red text). The tree was built with IQ-TREE version 1.6.10 (http://www.iqtree.org) using the best fit model indicated by the Model Finder with 1,000 bootstrap replicates. Text at nodes indicates bootstrap values >70. Brackets to the right indicate clades. Scale bar indicates number of nucleotide substitutions per site.

Despite the massive number of persons with SARS-CoV-2, only a few cases of active infection in pets have been reported (*3*). SARS-CoV-2–specific antibodies in pets have been reported on a few occasions, and higher seroprevalence rates have been found in animals from households in which family members have COVID-19 (*4*,*6*,*8*). The scarce reports of natural infection in dogs reflect their low susceptibility to SARS-CoV-2; for this infection, dogs are asymptomatic, produce limited titers, and have a reduced duration of viral shedding (*9*). Upon experimental infection, dogs shed SARS-CoV-2 at lower titers and for a shorter period than cats (*10*). Patterson et al. (*4*) found no actively infected dog or cat in a sampled population of 494 pets, including 67 dogs from households in which family members had COVID-19; however, SARS-CoV-2–specific antibodies were detected in a small proportion of pets (*4*). Delayed sampling of animals, caused by restrictions on human and animal movement during the pandemic, probably contributed to the negative results of molecular testing in that study. The infected poodle we report was monitored after the identification of the index case in the family, enabling the detection of SARS-CoV-2 RNA in swab samples collected during the observational follow-up. Because the canine virus shared 100% nucleotide identity with the virus detected in the index case, we believe human-to-dog transmission of the virus probably occurred in the household. 

## References

[1] Lorusso A, Calistri P, Petrini A, Savini G, Decaro N. Novel coronavirus (SARS-CoV-2) epidemic: a veterinary perspective. Vet Ital. 2020;56:5–10. 10.12834/VetIt.2173.11599.1 32048818

[2] Decaro N, Lorusso A. Novel human coronavirus (SARS-CoV-2): A lesson from animal coronaviruses. Vet Microbiol. 2020;244:108693. 10.1016/j.vetmic.2020.108693 32402329 PMC7195271

[3] Bosco-Lauth AM, Hartwig AE, Porter SM, Gordy PW, Nehring M, Byas AD, et al. Experimental infection of domestic dogs and cats with SARS-CoV-2: Pathogenesis, transmission, and response to reexposure in cats. Proc Natl Acad Sci U S A. 2020;117:26382–8.Error! Hyperlink reference not valid. 10.1073/pnas.2013102117 32994343 PMC7585007

[4] Patterson EI, Elia G, Grassi A, Giordano A, Desario C, Medardo M, et al. Evidence of exposure to SARS-CoV-2 in cats and dogs from households in Italy. Nat Commun. 2020;11:6231. 10.1038/s41467-020-20097-0 33277505 PMC7718263

[5] Corman VM, Landt O, Kaiser M, Molenkamp R, Meijer A, Chu DK, et al. Detection of 2019 novel coronavirus (2019-nCoV) by real-time RT-PCR [Erratum in: Euro Surveill. 2020;25: 20200409c] [Erratum in: Euro Surveill. 2020;25:2007303]. Euro Surveill. 2020;25:2000045. 10.2807/1560-7917.ES.2020.25.3.2000045 PMC6988269 31992387

[6] Zhang Q, Zhang H, Gao J, Huang K, Yang Y, Hui X, et al. A serological survey of SARS-CoV-2 in cat in Wuhan. Emerg Microbes Infect. 2020;9:2013–9. 10.1080/22221751.2020.1817796 32867625 PMC7534315

[7] Di Giallonardo F, Duchene S, Puglia I, Curini V, Profeta F, Cammà C, et al. Genomic epidemiology of the first wave of SARS-CoV-2 in Italy. Viruses. 2020;12:1438. 10.3390/v12121438 33327566 PMC7765063

[8] Fritz M, Rosolen B, Krafft E, Becquart P, Elguero E, Vratskikh O, et al. High prevalence of SARS-CoV-2 antibodies in pets from COVID-19+ households. One Health. 2021;11:100192. 10.1016/j.onehlt.2020.100192 33169106 PMC7641531

[9] Sit THC, Brackman CJ, Ip SM, Tam KWS, Law PYT, To EMW, et al. Infection of dogs with SARS-CoV-2. Nature. 2020;586:776–8. 10.1038/s41586-020-2334-5 32408337 PMC7606701

[10] Shi J, Wen Z, Zhong G, Yang H, Wang C, Huang B, et al. Susceptibility of ferrets, cats, dogs, and other domesticated animals to SARS-coronavirus 2. Science. 2020;368:1016–20. 10.1126/science.abb7015 32269068 PMC7164390

